# Shared names, different diseases: concordance of Thai traditional *Tor* diagnoses with biomedical eye conditions and back skin lesions

**DOI:** 10.3389/fmed.2026.1859102

**Published:** 2026-08-17

**Authors:** Piriya Chonsut, Paratthakorn Sangkaew, Weeratian Tawanwongsri, Wararee Sriyuttagrai, Pirada Yincharoen, Rini Sulastiwaty, Auemphon Mordmuang, Lunla Udomwech

**Affiliations:** 1 Department of Applied Thai Traditional Medicine, School of Medicine, Walailak University, Nakhon Si Thammarat, Thailand; 2 Center of Excellence in Tropical Pathobiology, Walailak University, Nakhon Si Thammarat, Thailand; 3 Department of Dermatology, School of Medicine, Walailak University, Nakhon Si Thammarat, Thailand; 4 Walailak University Hospital, Walailak University, Nakhon Si Thammarat, Thailand; 5 Department of Ophthalmology, School of Medicine, Walailak University, Nakhon Si Thammarat, Thailand; 6 Department of Surgery, School of Medicine, Walailak University, Nakhon Si Thammarat, Thailand; 7 Mayapada Eye Center, Jakarta, Indonesia; 8 Department of Medical Sciences, School of Medicine, Walailak University, Nakhon Si Thammarat, Thailand

**Keywords:** back nevi, diagnostic agreement, integrative medicine, nosology, ophthalmology

## Abstract

**Background:**

In Thailand, many eye diseases are described under the umbrella term *tor*, originating from Thai Traditional Medicine (TTM), including pinguecula, pterygium, cataract, and glaucoma (torlom, tornuea, torkrajok, and torhin). Ophthalmologists often use these terms as labels for biomedical diagnoses, whereas TTM practitioners use them within a traditional framework that includes characteristic back skin lesions. Apparent disagreement between diagnoses may therefore reflect nosologic mismatch rather than simple error. This study aimed to compare TTM tor diagnoses with ophthalmic diagnoses and to examine the association between tor categories and back skin lesion types.

**Methods:**

We conducted a double-blind study in adults attending a university ophthalmology clinic and an affiliated TTM clinic. All participants underwent ophthalmic examination and independent assessment by an ophthalmologist and a TTM practitioner. Back skin lesions identified as *tor*-related were photographed with dermoscopy and classified by a dermatologist. Agreement between TTM and ophthalmic diagnoses was evaluated using Cohen’s kappa, and the association between TTM diagnosis and lesion type was tested with Fisher’s exact test.

**Results:**

Of 140 enrolled participants, 128 were included in the ophthalmic analysis. Tornuea showed moderate agreement with pterygium (*κ* = 0.598) and torhin with glaucoma (*κ* = 0.407), whereas torlom and torkrajok showed no agreement with pinguecula or cataract. Among 186 skin lesions, there was no significant association between TTM diagnosis and dermatologic lesion type.

**Conclusion:**

TTM tor diagnoses therefore only partly overlap with biomedical eye disease categories, and *tor*-related back lesions appear to be benign rather than disease-specific markers.

## 1 Introduction

**Figure 1:**
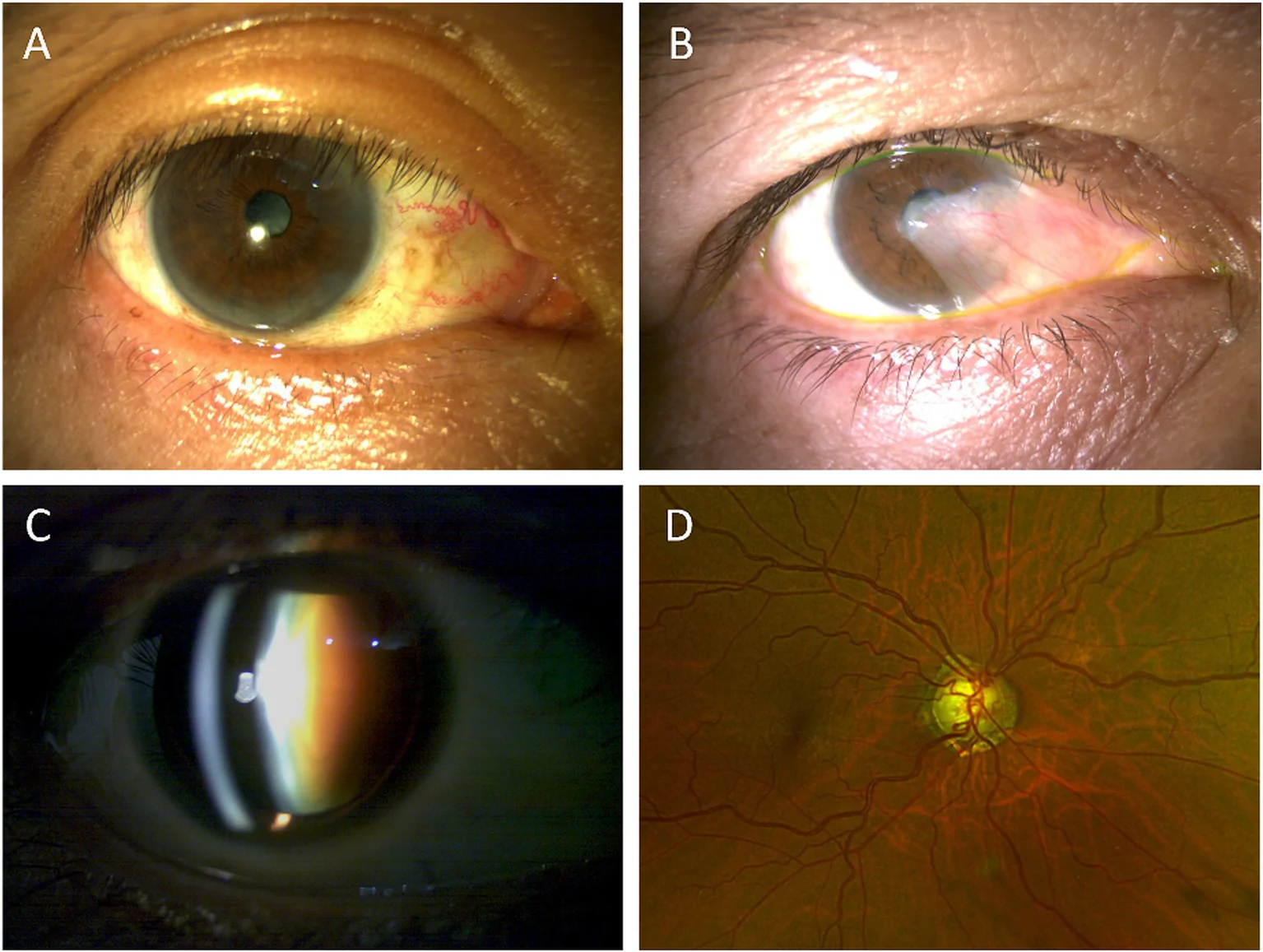
Representative ophthalmic photographs of **(A)** pinguecula, **(B)** pterygium, **(C)** cataract, and **(D)** glaucoma.

Cataract, glaucoma, and degenerative ocular surface diseases such as pinguecula and pterygium are among the most common causes of visual impairment in Thailand, particularly in older adults and people with high exposure to sunlight, dust, and wind (1–3). In everyday Thai, these conditions are grouped under the generic term *tor* (ต้อ /tɔ̂:/), with more specific labels such as torlom (ต้อลม /tɔ̂ː lom/): pinguecula; tornuea (ต้อเนื้อ /tɔ̂ː nɯ́a/): pterygium; torkrajok (ต้อกระจก /tɔ̂ː kràʔ tɕòk/): cataract; and torhin (ต้อหิน /tɔ̂ː hǐn/): glaucoma (Figure 1). Ophthalmologists commonly use these tor terms when explaining diagnoses to patients, even though the words themselves long predate the arrival of biomedical ophthalmology and are rooted in Thai Traditional Medicine (TTM) concepts (4, 5).

Classical Thai Traditional Medicine (TTM), particularly the Abhayasuntara scripture, describes a substantially broader classification of eye diseases than the four contemporary *tor* categories evaluated in this study. In addition to torlom, tornuea, torkrajok, and torhin, the classical text describes numerous other tor entities, including tortapu, torkongkwian, torninlapat, torsalak, tormok, and torlin sunakh, as well as several subtypes of torkrajok. These traditional entities are distinguished by combinations of ocular manifestations, systemic signs, disease origins, and diagnostic patterns rather than by direct correspondence to biomedical disease categories. In contemporary Thai healthcare, however, the terms torlom, tornuea, torkrajok, and torhin are widely used in communication between TTM practitioners, ophthalmologists, and patients. Accordingly, the present study focused on these four contemporary tor categories because they represent the shared diagnostic terminology most commonly encountered in integrative clinical practice.

**Figure 2:**
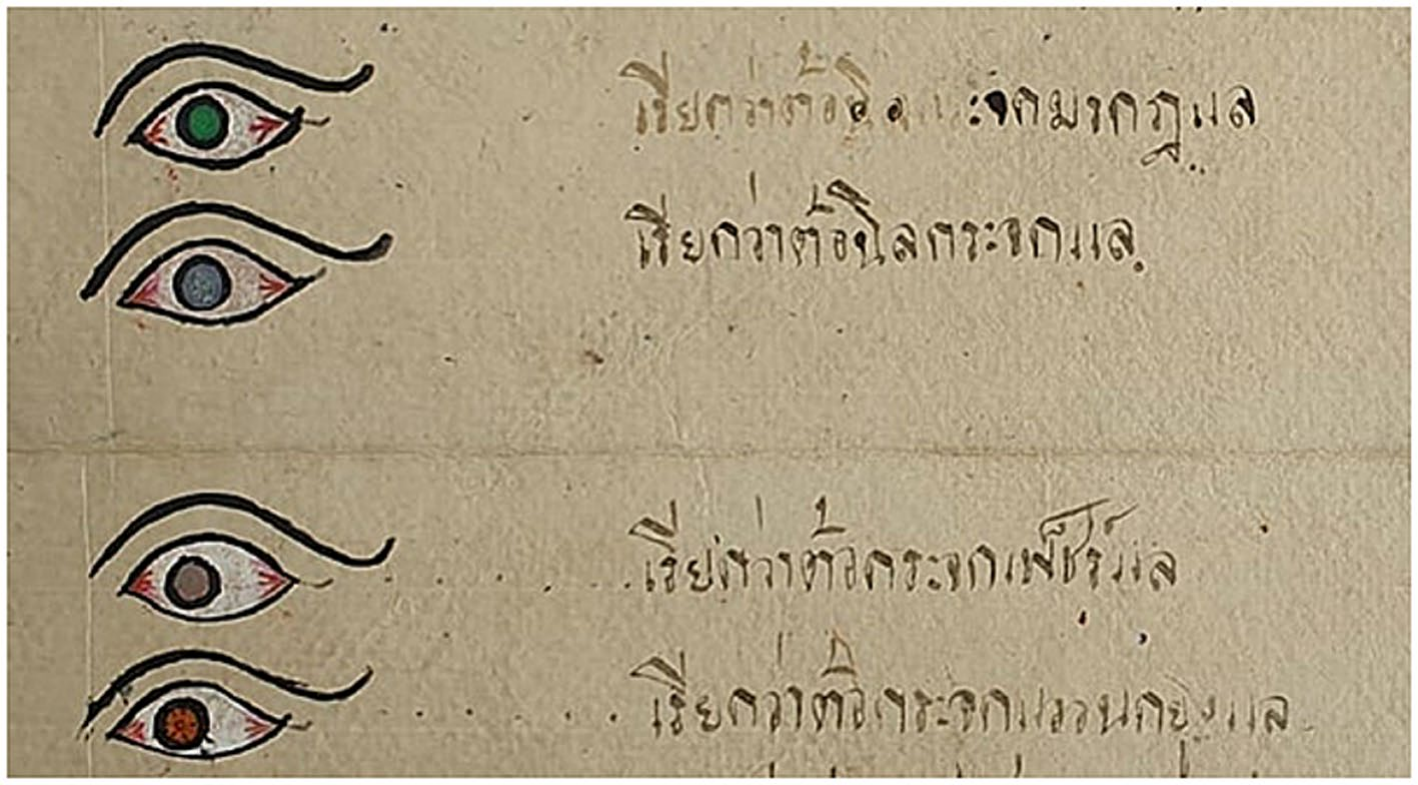
Abhayasuntara TTM scripture describing eye diseases. The illustrated eyes are subtypes of cataract or T*orkrajok* in the TTM concept.

The conceptual foundations of TTM and biomedical ophthalmology, however, are markedly different. TTM interprets illness through imbalances of the elemental qualities (earth, water, wind, and fire) and diagnostic patterns based on systemic signs, pulse examination, and body-element assessment, whereas modern ophthalmology defines disease entities according to tissue pathology, structural changes, and functional impairment (4–6). When traditional and biomedical medicine both use the terms torlom, tornuea, torkrajok, and torhin, it is often implicitly assumed that these labels refer to the same disease entities. However, preliminary review of classical TTM scriptures (Figure 2) and contemporary clinical practice suggests that the symptom complexes and pathophysiological concepts associated with these tor terms in TTM may differ substantially from their biomedical counterparts, raising the possibility that different disease constructs are being described using the same terminology (6, 7). Accordingly, the present study does not assume a one-to-one equivalence between TTM and biomedical diagnoses but instead evaluates how these commonly used diagnostic labels correspond in contemporary clinical practice.

**Figure 3:**
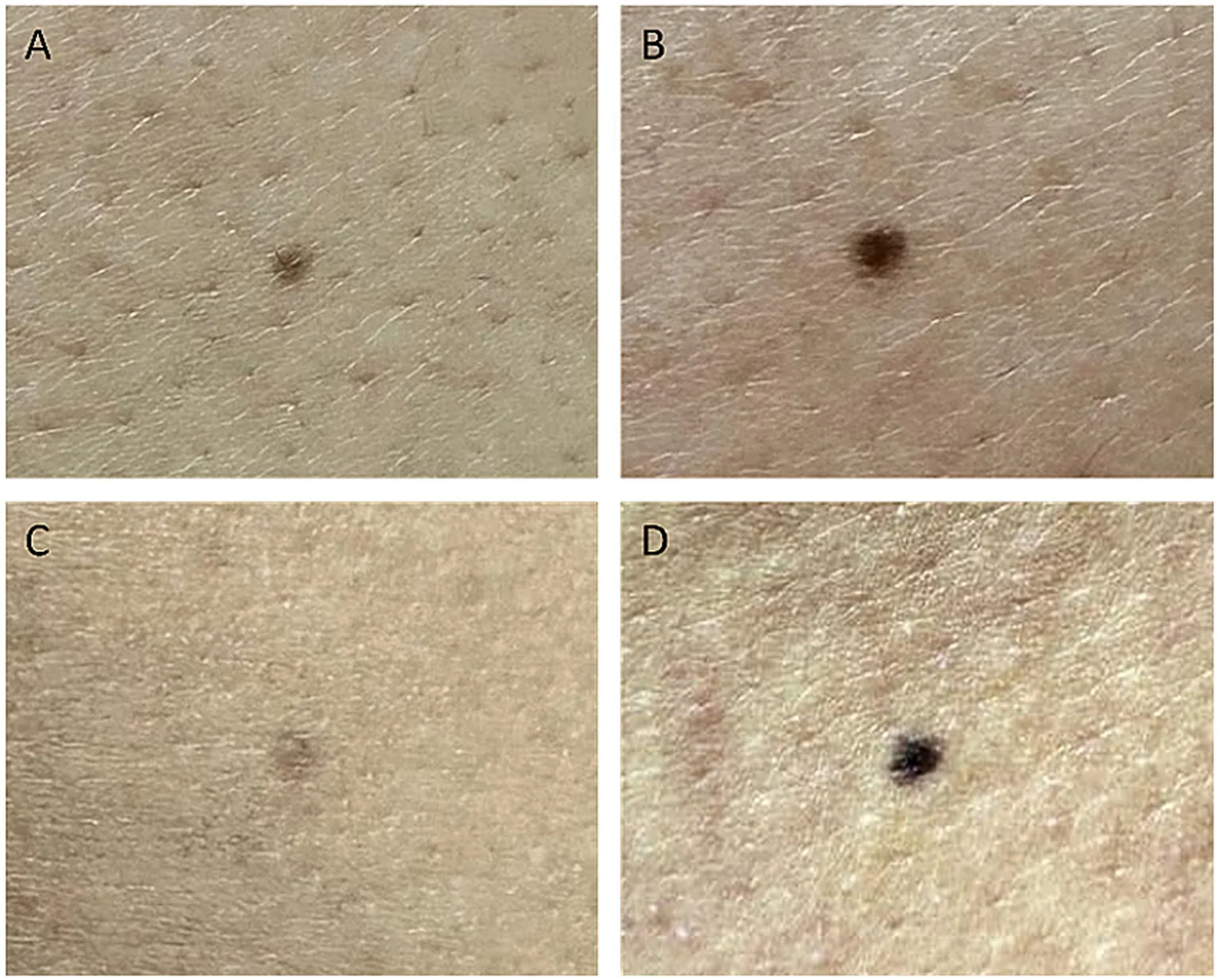
Pigmented lesions, *tor*, found on backs of eye disease patients, according to Thai Traditional Medicine concept. Panels **(A–D)** illustrate *tor* lesions traditionally interpreted as with pinguecula *torlom*, pterygium *tornuea*, cataract *torkrajok*, and glaucoma *torhin*, respectively.

For TTM practitioners, tor is not confined to ocular findings, but eye diseases are believed to exhibit characteristic skin lesions on the back, with distinct patterns corresponding to different eye diseases. According to the TTM diagnostic framework, each *tor* eye disease is characterized by a distinct combination of ocular symptoms and characteristic back skin lesions. These lesions are interpreted as part of the overall diagnostic pattern rather than as isolated dermatologic abnormalities. Patients diagnosed with torkrajok are traditionally described as having blurred vision accompanied by a transparent shadow or haze within the eye. Examination of the back is expected to reveal a small, flat, black or brown pigmented lesion measuring approximately 1–3 mm in diameter, with a pale central area. This lesion is traditionally referred to as the “torkrajok point.” Patients with torlom typically present with ocular irritation, itching, and excessive tearing. Eye examination reveals a white or yellowish lesion on the nasal conjunctiva. The corresponding back findings are described as multiple small, flat, light brown pigmented lesions measuring approximately 1–3 mm in diameter. Patients with tornuea commonly experience ocular irritation, itching, and tearing, with symptoms becoming more pronounced during episodes of inflammation. Eye examination reveals a flesh-like tissue extending from caruncle onto the cornea, most frequently on the nasal side. According to TTM, examination of the back reveals small, flat, dark brown or black pigmented lesions measuring approximately 1–3 mm in diameter. Patients with torhin are traditionally described as having blurred vision, with the perception of a whitish opacity over the pupil. The associated back findings are described as numerous small, flat, dark black pigmented lesions measuring approximately 1–3 mm in diameter. Compared with the other tor categories, these lesions are traditionally considered to exhibit the darkest pigmentation (Figure 3).

In TTM clinical practice, these back lesions are used as a principal diagnostic guide, in conjunction with eye examination and history taking. Manipulation of these lesions (for example, pressing, scraping, or needling) is believed to contribute to the treatment or cure of eye disease (4, 5). From a biomedical perspective, these lesions may represent a range of dermatologic conditions rather than disease-specific markers. In this study, a dermatologist systematically evaluates and classifies the back skin lesions identified by TTM practitioners into standard dermatologic lesion types, providing a bridge between TTM pattern recognition and modern skin disease taxonomy (8, 9).

Although this nosologic mismatch may seem like a problem of terminology, it can have practical consequences in clinical care. An example scenario is when patients seek a second opinion, moving from a TTM clinic to an ophthalmology clinic, or from an ophthalmology clinic to a TTM practitioner-ophthalmologists and TTM practitioners may appear to dispute each other’s diagnoses or treatments, when in fact they are diagnosing different diseases that share the same tor label. Apparent “misdiagnosis” or “wrong treatment” from the perspective of one system may simply reflect a different disease construct in the other (6, 7). For patients, the reuse of historically TTM-based terminology by modern physicians can further blur boundaries: a person told they have torlom or torhin by different providers may assume continuity of diagnosis where none exists or interpret changes in diagnostic labels as professional disagreement rather than a consequence of divergent frameworks.

In this context, we designed a double-blind study in which ophthalmologists, TTM practitioners, and dermatologists independently assess the same set of patients. The primary aim is to determine whether torlom, tornuea, torkrajok, and torhin, as diagnosed in routine TTM practice, correspond to the same clinical entities as pinguecula, pterygium, cataract, and glaucoma diagnosed by ophthalmologists, or whether these shared labels in fact refer to different diseases in the same patients. The secondary aim is to systematically describe and classify the back skin lesions used by TTM practitioners in diagnosing *tor* diseases by characterizing their morphology as dermatologic lesion types and to explore how these lesions relate to *tor* diagnoses in both systems. By clarifying whether the diseases behind the shared *tor* terminology are truly the same, and by interpreting TTM back lesions using dermatologic terminology, this study aims to provide an empirical basis for safer communication, fewer inter-professional disputes, and more conceptually coherent integration of traditional and modern eye care in Thailand (7).

## 2 Materials and methods

This study employed a double-blind comparative descriptive design to compare diagnoses made independently by ophthalmologists, TTM practitioners, and a dermatologist. The dermatologist was blinded to both ophthalmologic and TTM diagnoses. Participants were enrolled between January and October 2025 from the Ophthalmology Clinic, Walailak University Hospital, and the Applied Thai Traditional Medicine Clinic, Walailak University Hospital. Adults aged ≥ 18 years who attended either clinic, agreed to participate voluntarily, and provided written informed consent after receiving an explanation of the study objectives and procedures were eligible for inclusion. Participants who declined to provide information, withdrew consent during the study, or were unable to complete both the ophthalmic and TTM examinations were excluded.

Although classical TTM describes a broader range of tor eye diseases, the present study focused on four contemporary tor categories (torlom, tornuea, torkrajok, and torhin) because these were selected for comparison with the corresponding biomedical eye diseases within the study design.

The sample size was calculated using statistical software with the following parameters: minimum acceptable kappa = 0.6, expected kappa (*κ*) = 0.8, proportion of outcome (*p*) = 0.5, significance level (*α*) = 0.05, statistical power (1 − *β*) = 80%, and an anticipated dropout rate of 10% (10, 11). Based on these parameters, the required sample size was 126 participants, with an adjusted target of 140 participants to account for attrition.

The sample size (*n*) was computed using the following equation:

$$n={\left(\frac{{\mathrm{sd}}_{0}.z_{1-\alpha }+{\mathrm{sd}}_{1}.z_{1-\beta }}{K_{1}-K_{0}}\right)}^{2}$$

K₀ = minimum acceptable Cohen’s kappa under the null hypothesis (0.60).

K₁ = expected Cohen’s kappa under the alternative hypothesis (0.80).

α = Type I error probability (0.05).

β = Type II error probability (0.20).

z₁−α = standard normal deviate corresponding to α.

z₁−β = standard normal deviate corresponding to statistical power.

sd₀ = standard deviation of *κ* under the null hypothesis.

sd₁ = standard deviation of *κ* under the alternative hypothesis.

### 2.1 Data collection process

#### 2.1.1 Recruitment

The study was publicized at both participating hospitals. Patients who had interest and met the inclusion criteria were invited to participate and provided informed consent.

#### 2.1.2 Initial diagnosis

Each participant underwent history taking and physical examination by either an ophthalmologist (L. U. or W. S.) or an applied TTM practitioner (P. C. or P. S.). Clinical symptoms, signs, and diagnostic findings were recorded in standardized forms.

The TTM assessments were performed independently by experienced licensed Thai Traditional Medicine practitioners (P. C. and P. S.) with formal training in Thai Traditional Medicine and clinical experience in the diagnosis and management of ophthalmic TTM disorders of more than five years. Both practitioners routinely provide TTM clinical services at the Applied Thai Traditional Medicine Hospital, Walailak University, and perform diagnostic assessments according to established TTM principles, including the interpretation of *Tor* eye diseases based on classical Thai Traditional Medicine concepts.

#### 2.1.3 Ophthalmic evaluation

All patients received full ophthalmic examinations and history interviewing, including visual acuity, intraocular pressure (Nidek non-contact tonometer, NT-530, Japan), anterior segment examinations with fluorescein staining, and fundoscopic examinations (Haag-Streit slit lamp biomicroscope, BT-900, Switzerland). Presence of any ocular disease is noted. Pinguecula is graded 0–2; pterygium is graded 0–3; and cataract is graded using LOCS III criteria consisting of nuclear color (NC) and opalescence (NO) (grade 1–6), cortical cataract (C) (grade 1–5), and posterior subcapsular cataract (P) (grade 1–5) (12).

#### 2.1.4 Cross-diagnostic evaluation

Participants were then referred for complementary diagnosis: those initially examined by ophthalmologists were further evaluated by TTM practitioners, and vice versa. Each practitioner completed their assessment independently and was blinded to the other’s diagnosis.

#### 2.1.5 Identification of back skin lesions

The TTM practitioner identified characteristic tor lesion(s) on the patients’ backs, corresponding to sites associated with eye function in TTM. These lesions were marked and photographed using a dermoscope (10 × magnification) to capture skin surface morphology. The lesions were circled with a non-permanent white marker to enable precise localization and photographed with a dermoscope (DermLite® DL5) for both diagnostic and documentation purposes. Standardized clinical photographs of the lesions were taken (Figure 1). The lesions were grouped according to established dermoscopic criteria, including lentigo simplex/nevus, seborrheic keratosis, basal cell carcinoma, vascular lesions, dermatofibroma, squamous cell carcinoma, melanoma, or other categories (8, 9). When all procedures had been completed, the temporary markings were gently removed with normal saline.

The recorded variables included: (1) clinical diagnosis by the TTM practitioner (torlom, tornuea, torkrajok, torhin); (2) lesion color (black, brown, gray, or blue); (3) palpability (yes or no); and (4) final dermatologic diagnosis confirmed by the dermatologist.

When evaluated according to biomedical dermatologic criteria, these lesions were predominantly common benign conditions, including nevi, seborrheic keratoses, and solar lentigines. Their dermatologic classification did not differ significantly among the four TTM ‘*tor’* categories. These findings indicate that, from a biomedical dermatologic perspective, lesion type alone is not a disease-specific marker for the corresponding ophthalmic diagnoses. Within the TTM framework, however, the diagnostic significance of these lesions is based on a broader pattern-recognition system that incorporates lesion location, morphology, associated symptoms, and other traditional diagnostic findings.

#### 2.1.6 Dermoscopic assessment

The dermoscopic images were independently reviewed by a board-certified dermatologist (W. T.), who confirmed the clinical diagnoses of the back lesions and described cutaneous characteristics such as pigmentation, vascularity, and texture according to current dermoscopic criteria (8, 9).

### 2.2 Statistical analysis

The research team compiled and compared diagnostic data from ophthalmologists, TTM practitioners, and dermatologist to assess the consistency and relationships between traditional and modern diagnostic findings.

The level of agreement between diagnoses made by ophthalmologists and TTM practitioners was analyzed using Cohen’s kappa coefficient (*κ*) (10, 11). Correlations were assessed not only between translational counterparts (for example pinguecula–torlom), but also across all combinations of conventional and TTM diagnoses, given the possibility of incorrect nosology.

The association between TTM diagnoses and dermatologic lesion types (nevus, seborrheic keratosis, solar lentigo) was analyzed using chi-square tests and Fisher’s exact test with Monte Carlo estimation, because several cells had expected counts < 5 (10). A *κ* value above 0.60 was interpreted as substantial agreement, and a *p*-value < 0.05 was considered statistically significant (10, 11). Statistical analyses were performed using IBM SPSS Statistics version 23.0 (IBM Corp., Armonk, NY, USA).

## 3 Results

The research was conducted over a 12-month period, beginning after ethical approval and encompassing participant recruitment, clinical evaluation, dermoscopic imaging, and statistical analysis. Of the 140 participants, eight were eliminated due to missing data. Demographic data is shown in Table 1. These patterns are compatible with the age-related burden of cataract, glaucoma, and ocular surface disease reported in other populations (1–3).

**Table 1:** Baseline demographic characteristics and underlying diseases of the study participants (*n* = 128).

		Mean	SD	Range
Age	Mean	61.20	±14.29	19–88
		N	Percent	
Sex	Male	42	32.80	
	Female	86	67.20	
Underlying diseases	None	29	22.66	
	Hypertension	41	32.03	
	Diabetes mellitus type 2	29	22.66	
	Dyslipidemia	49	38.28	
	Others	37	28.91	
Total cases		128		

### 3.1 Agreement between TTM and ophthalmic diagnoses

Correlation between biomedical and TTM ophthalmic diagnoses was assessed for both matched and off-diagonal pairs (Table 2). Across the four biomedical ophthalmic diagnoses and their TTM counterparts, pinguecula and torlom showed a small-to-moderate positive association (*κ* = 0.266, *p* = 0.001), pterygium demonstrated moderate agreement with tornuea (*κ* = 0.598, *p* < 0.001), cataract showed a non-significant correlation with torkrajok (*κ* = −0.046, *p* = 0.587), and glaucoma exhibited moderate agreement with torhin (*κ* = 0.407, *p* < 0.001); however, the torhin subgroup comprised only nine lesions; therefore, these findings should be interpreted cautiously. Off-diagonal comparisons suggested limited cross-mapping between TTM and conventional labels. Pinguecula showed a small negative association with torkrajok (*κ* = −0.159, *p* = 0.004), and pterygium showed a weak negative association with torlom (*κ* = −0.071, *p* = 0.049), implying that these TTM terms were not being used interchangeably with other ocular surface diagnoses. Glaucoma showed a small negative association with torkrajok (*κ* = −0.171, *p* = 0.033). Cataract did not demonstrate a statistically significant association with any of the four TTM categories, suggesting that the TTM label torkrajok may not consistently capture the biomedical construct of cataract within this cohort. Overall, these patterns support selective rather than universal concordance between the two systems, with the clearest alignment observed for tornuea-pterygium and torhin-glaucoma, and weaker or absent alignment for torlom–pinguecula and torkrajok-cataract.

**Table 2:** Concordance between ophthalmic diagnoses (pinguecula, pterygium, cataract, and glaucoma) and Thai Traditional Medicine diagnoses (torlom, tornuea, torkrajok, and torhin) assessed using Cohen’s kappa coefficient.

TTM diagnoses Ophthalmic diagnoses	Torlom	Tornuea	Torkrajok	Torhin
Pinguecula	*κ* = 0.266 *p* = 0.001*	*κ* = 0.052 *p* = 0.294	*κ* = −0.159 *p* = 0.004*	*κ* = 0.049 *p* = 0.072
Pterygium	*κ* = −0.071 *p* = 0.049*	*κ* = 0.598 *p* < 0.001*	*κ* = −0.081 *p* = 0.359	*κ* = −0.124 *p* = 0.067
Cataract	*κ* = −0.033 *p* = 0.551	*κ* = 0.120 *p* = 0.142	*κ* = −0.046 *p* = 0.587	*κ* = −0.046 *p* = 0.337
Glaucoma	*κ* = 0.040 *p* = 0.107	*κ* = 0.011 *p* = 0.892	*κ* = −0.171 *p* = 0.033*	*κ* = 0.407 *p* < 0.001*

*κ*, Cohen’s kappa coefficient; *p-values indicate the statistical significance of agreement between diagnostic methods. *p* < 0.05 indicates statistical significance.

*κ* < 0 Less than chance agreement (poor).

*κ* 0.01–0.20 None to slight agreement.

*κ* 0.21–0.40 Fair agreement.

*κ* 0.41–0.60 Moderate agreement.

*κ* 0.61–0.80 Substantial agreement.

*κ* 0.81–1.00 Almost perfect agreement.

### 3.2 Association between TTM diagnoses and dermatologic lesion types

A total of 186 back skin lesions were documented and classified as nevus, seborrheic keratosis, or solar lentigo. Their distribution by TTM diagnosis was as follows. Among the 186 documented back skin lesions, torlom was associated with 102 nevi, 5 seborrheic keratoses, and 6 solar lentigines (113 lesions in total). Tornuea corresponded to 27 nevi and 2 seborrheic keratoses, with no solar lentigines (29 lesions). Torkrajok was linked to 33 nevi and 2 solar lentigines, without any seborrheic keratoses (35 lesions), while torhin was associated with 8 nevi and 1 seborrheic keratosis, and no solar lentigines (9 lesions) (Table 3). Chi-square tests and Fisher’s exact test with Monte Carlo estimation showed no significant association between TTM diagnosis and dermatologic lesion type (Fisher’s exact test with Monte Carlo estimation, *p* = 0.45). Nevi, seborrheic keratoses, and solar lentigines were similarly distributed across torlom, tornuea, torkrajok, and torhin (Table 4).

**Table 3:** Dermatologic classification of back lesions corresponding TTM *“tor”* eye disease diagnoses.

		Dermatologic diagnoses			Total
		Nevus	Seb K	Solar lentigines	
TTM diagnoses	Torlom	102	5	6	113
	Tornuea	27	2	0	29
	Torkrajok	33	0	2	35
	Torhin	8	1	0	9
Total		170	8	8	186

A single patient may be present with more than one dermatologic diagnosis.

**Table 4:** Association between TTM *“tor”* diagnoses and dermatologic diagnoses analyzed using Chi-square and Fisher’s exact tests.

	Value	df	Asymptotic significance (2-sided)	Monte Carlo Sig. (2-sided)			Monte Carlo Sig. (1-sided)		
				Significance	99% Confidence interval		Significance	99% Confidence interval	
					Lower bound	Upper bound		Lower bound	Upper bound
Pearson Chi-Square	5.061^a^	6	0.536	0.495^b^	0.482	0.508			
Likelihood Ratio	7.798	6	0.253	0.336^b^	0.324	0.348			
Fisher’s Exact Test	4.907			0.452^b^	0.440	0.465			
Linear-by-Linear Association	.323^c^	1	0.570	0.607^b^	0.594	0.619	0.327^b^	0.315	0.339
N of Valid Cases	186								

^a^8 cells (66.7%) have expected count less than 5. The minimum expected count is 0.39.

^b^Based on 10,000 sampled tables with starting seed 2000000.

^c^The standardized statistic is −0.569.

## 4 Discussion

### 4.1 Principal findings

Although the cross-sectional design precludes assessment of temporal or causal relationship, this double-blind study showed that only some TTM *tor* diagnoses correspond closely to biomedical eye diseases. Tornuea had moderate agreement with pterygium, and torhin had moderate agreement with glaucoma. Torlom showed only small-to-moderate agreement with pinguecula, and torkrajok had no meaningful association with cataract. Negative off-diagonal *κ* values suggested that TTM practitioners were not simply shifting *tor* labels to other modern eye diagnoses.

For the skin findings, there was no significant association between TTM diagnosis (torlom, tornuea, torkrajok, torhin) and the type of back skin lesion (nevus, seborrheic keratosis, or solar lentigo). The distribution of lesion types was similar across all four TTM groups.

### 4.2 Nosology and overlap between TTM and biomedical diagnoses

Our results address the main question of this study: when TTM practitioners and ophthalmologists use the same *tor* words, are they describing the same diseases?

The data suggests a mixed picture. For tornuea-pterygium and torhin-glaucoma, the level of agreement supports the usual clinical assumption that these pairs often refer to similar conditions, consistent with the clinical and epidemiological profile of pterygium and glaucoma in aging, sun-exposed populations (1–3). In contrast, torlom and pinguecula matched only weakly, and torkrajok did not match cataract at all. This indicates that torkrajok, as used in current TTM practice, is not simply the TTM word for cataract, but may represent a broader or different disease concept.

Another possible explanation is that TTM practitioners may recognize *Torkrajok* primarily in more advanced stages of cataract, whereas biomedical ophthalmology identifies cataracts across a wider spectrum of severity, including early lens opacities detectable by slit-lamp examination and LOCS III grading. The present study was not designed to evaluate the influence of cataract severity on TTM diagnosis. Future studies stratifying patients according to cataract severity may help clarify whether diagnostic concordance changes across different stages of disease.

Overall, the use of the same Thai disease names in TTM and modern medicine does not necessarily indicate that both systems refer to identical disease entities. Rather, these terms may represent different diagnostic constructs within distinct medical frameworks. This has practical implications when clinicians communicate across systems, record diagnoses in medical records, or develop integrative care pathways (6, 7, 13, 14). It also highlights the importance of interpreting disease labels within their respective epistemological frameworks rather than assuming direct one-to-one equivalence between TTM and biomedical diagnoses.

### 4.3 Assessment of the back skin lesions in TTM and in dermatology

TTM practitioners in this study routinely used back skin lesions as a key part of diagnosis for *tor* eye diseases and believed that manipulating these lesions can help treat eye problems. From the TTM perspective, these lesions are closely connected to eye function and to imbalance of the elements (4, 5).

When these lesions were examined by a dermatologist and categorized using dermoscopy, most were classified as common benign conditions, mainly nevi, seborrheic keratoses, and solar lentigines (8, 9). The dermatologic classification of these lesions did not differ significantly among torlom, tornuea, torkrajok, and torhin. These findings indicate that, from a biomedical dermatologic perspective, lesion type alone was not associated with specific ophthalmic diagnoses in this cohort. Rather, the lesions represented common benign dermatologic conditions that have diagnostic significance within the TTM framework. It should be noted that these benign dermatologic classification of these lesions does not negate their diagnostic significance within the TTM framework, which is based on pattern recognition and systemic imbalance rather than local tissue pathology.

These findings do not diminish the cultural or clinical importance of back lesions in TTM practice. Instead, they suggest that the diagnostic significance of these lesions lies within the epistemological framework of TTM, in which lesion location, morphology, associated symptoms, and systemic diagnostic patterns are interpreted together rather than as isolated dermatologic abnormalities. In contrast, the present study evaluated only the dermatologic classification of these lesions according to biomedical criteria. Therefore, the absence of an association between dermatologic lesion type and TTM diagnosis should not be interpreted as a judgment on the theoretical validity of TTM. Rather, it reflects differences between two distinct diagnostic systems that use different conceptual frameworks to interpret clinical findings.

### 4.4 Implications for patient care, communication, and integrative services

These findings help explain why conflict sometimes appears when patients seek a second opinion between TTM and ophthalmology clinics. A patient may be told that they have torkrajok by a TTM practitioner but be told that they have no significant cataract by an ophthalmologist. Without understanding the different disease concepts, both sides may feel that the other is “wrong,” and the patient may lose trust in one or both systems. Our results suggest that many of these disagreements are due to different meanings hidden behind the same words, rather than simple diagnostic error, echoing earlier work on divergent “illness” and “disease” models in cross-cultural medicine (6). For clinical practice, ophthalmologists and dermatologists who use *tor* terminology should be aware that patients and TTM colleagues may attach a wider TTM-based meaning to these terms, including expectations about back lesions and their treatment. This is particularly relevant where national and international policy frameworks encourage integration of traditional medicine within universal health coverage (7, 13, 14). Communication quality and health literacy are also likely to influence how patients interpret such overlapping terms (15, 16). Patients with limited health literacy may assume that the same *tor* label necessarily indicates the same disease, even when clinicians from different systems are using that label in different ways. In Thailand, recent work has shown that health literacy plays a key role in how people use and understand TTM-related information, including self-care practices (17). Our findings add that the very words used for diagnosis can themselves be a source of confusion. Simple communication strategies could help, such as explaining which biomedical diagnosis is meant when using a *tor* word; stating clearly when there is no exact one-to-one match (especially for torkrajok–cataract); and using referral notes that list both the *tor* term and the corresponding modern diagnosis or explicitly state that no clear counterpart exists, thus referral documents may include both biomedical and TTM diagnoses when appropriate. These steps may reduce misunderstanding, improve shared decision-making, and support safer integrative care (13, 14).

### 4.5 Implications for education and future research

For education, this study suggests that training programs for both TTM and biomedical professionals should address basic principles of cross-system diagnosis and medical language. Using concrete examples such as tornuea – pterygium and torkrajok – cataract can help trainees understand how a single Thai term can represent different clinical ideas, and why these matters in daily practice. This aligns with calls to integrate traditional, complementary, and alternative medicine topics into health professional curricula in a structured way (18).

Future research could build on this work in several ways. Qualitative studies with TTM practitioners and patients could explore in more depth how *tor* diseases and back lesions are understood, and how these beliefs influence treatment choices (6). Larger, multi-centre quantitative studies could test whether the agreement patterns seen here are similar in other regions or other TTM schools. It would also be valuable to investigate whether the location or pattern of back lesions, rather than lesion type alone, shows any consistent relationship with ocular or systemic risk factors.

Given that many of the patients who present to both TTM and ophthalmology clinics are older adults with multiple chronic conditions, this work should also be interpreted in the context of multimorbidity (19, 20). Future studies could examine how *tor* diagnoses fit within broader multi-disease profiles and whether they influence prioritization of care across systems.

### 4.6 Strengths and limitations

This study has several strengths. The double-blind design reduced bias between TTM practitioners, ophthalmologists, and the dermatologist. Standardized ophthalmic examinations and LOCS III grading improved the reliability of biomedical cataract diagnoses (12). Dermoscopic imaging and independent dermatologic review provided detailed and reproducible descriptions of the back lesions (8, 9). Using kappa statistics and Fisher’s exact test with Monte Carlo estimation allowed robust analysis despite some small cell counts (10, 11).

However, this study also has several limitations. First, it was conducted at a single university-affiliated center, which may not represent all TTM or ophthalmic practice patterns in Thailand. Second, only four contemporary *tor* categories were evaluated. Classical Thai Traditional Medicine, particularly the Abhayasuntara scripture, recognizes a substantially broader taxonomy of *tor* eye diseases and their subtypes. Consequently, reducing TTM diagnoses to these four categories may oversimplify the traditional nosological framework, and the findings should not be interpreted as representing the full spectrum of TTM ophthalmology. Third, some TTM categories contained relatively few participants, limiting statistical power for certain comparisons. Fourth, only clinically significant cataract (NS, NO, C, and *p* ≥ 3 by LOCS III) was counted as cataract in calculation; using different cut point might yielded different result. Fifth, back skin lesions were classified by a single dermatologist, and inter-observer agreement was not assessed. Finally, the study evaluated only three common benign dermatologic lesion types; other cutaneous findings described in TTM were beyond the scope of the present investigation.

## 5 Conclusion

This study shows that TTM *tor* diagnoses and biomedical eye disease categories overlap only partly. Tornuea and torhin correspond reasonably well to pterygium and glaucoma, whereas torlom matches pinguecula only weakly, and torkrajok showed poor agreement with cataract. Back skin lesions used by TTM practitioners as key diagnostic and therapeutic sites are common benign dermatologic lesions and are not specifically linked to any single eye disease. Back skin lesions identified by TTM practitioners were classified predominantly as common benign dermatologic lesions, and their biomedical dermatologic classifications were not specifically associated with individual ophthalmic diagnoses in this cohort.

Recognizing these differences can help clinicians understand apparent disagreements between systems, improve communication with patients, and design integrative eye-care services that respect both TTM and modern medicine without assuming that shared disease names always refer to the same clinical entity.

## Data Availability

The original contributions presented in the study are included in the article/supplementary material, further inquiries can be directed to the corresponding author.
